# Real-time monitoring system for evaluating the acid-producing activity of oral squamous cell carcinoma cells at different environmental pH

**DOI:** 10.1038/s41598-017-10893-y

**Published:** 2017-08-30

**Authors:** Hiromitsu Morishima, Jumpei Washio, Jun Kitamura, Yuta Shinohara, Tetsu Takahashi, Nobuhiro Takahashi

**Affiliations:** 1 Division of Oral Ecology and Biochemistry, Department of Oral Biology, Tohoku University Graduate School of Dentistry, Sendai, Japan; 2 Division of Oral and Maxillofacial Surgery, Tohoku University Graduate School of Dentistry, Sendai, Japan; 3 Division of Advanced Prosthetic Dentistry, Tohoku University Graduate School of Dentistry, Sendai, Japan

## Abstract

This study aimed to establish a real-time monitoring system for evaluating the acid-producing activity of cells and the effects of microenvironmental pH on their metabolism. Oral squamous cell carcinoma (HSC-2, HSC-3) and normal (HaCaT) cells were used. Their acid-producing activity from glucose, glutamine, and glutamate was monitored at various pH values using a pH stat system. Their production of lactic acid and ammonia was also measured. The acid-producing activity was monitored successfully. Both the cancer and normal cells produced acids from glucose, glutamine, and glutamate. All of the cells decreased their acid-producing activity as the environmental pH fell, but in glucose-derived acid-producing activity the cancer cells were more acid-tolerant than HaCaT cells. In the cancer cells, the proportion of lactic acid among all acids produced from glucose at the acidic environment tended to be higher than that in HaCaT cells. All of the cells produced ammonia from glutamine, while only HaCaT cells produced ammonia from glutamate. We established a real-time monitoring system for evaluating the acid-producing activity of cells. Our results suggest that the cancer cells possess acid-tolerant glucose metabolism with a tendency of metabolic shift to lactic acid production at acidic pH and they metabolise glutamate without ammonia production.

## Introduction

Many cancer-related genes, such as Myc and p53, have been reported^1, 2^, and it has become clear that the metabolic activity of cancer cells is regulated by these oncogenes^3^. The supply of energy and cell constituents is crucial for the infinite proliferation of cancer cells, and therefore, elucidating their metabolic systems might provide essential information about cancer cells.

It is well known that cancer cells exhibit a characteristic metabolic phenomenon called the Warburg effect^4^; i.e., they produce lactic acid from glucose even in the presence of abundant oxygen. Furthermore, it has been reported that cancer cells display enhanced glutamine metabolism, so-called glutaminolysis^5, 6^. We have confirmed that oral squamous cell carcinoma (OSCC) cells also demonstrate similar metabolic activity^7^. These observations suggest that the pH of the microenvironment around cancer cells tends to change in response to the levels of acidic and alkaline metabolic products, such as lactic acid and ammonia. Extracellular acidosis is also reported to be a feature of cancer cells^8, 9^, and so the microenvironmental pH of cancer cells is considered to be different from that of normal cells.

The relationships between environmental factors and cancer cells have been examined by many researchers, and it is becoming clear that environmental factors, such as acidic pH and low oxygen levels, are involved in the expression of genes, such as those encoding glucose transporter 1 and hexokinase II, through hypoxia-inducible factor-1^10, 11^, and the inhibition of the tricarboxylic acid (TCA) cycle via the expression of pyruvate dehydrogenase kinase 1, an enzyme responsible for the inhibition of pyruvate dehydrogenase^12^. Moreover, it has been suggested that cancer metastasis was increased in mice by acidic pH^13^, and in human head and neck cancer tissue a high concentration of lactic acid was found to increase the risk of metastasis^14^. It was also reported that a low pH microenvironment affected the permeability of a weakly alkaline drug and was associated with resistance to anticancer drugs^15^.

However, it remains unclear how environmental pH directly affects metabolic activity, probably because the biological activity of cancer cells has mainly been evaluated based on their proliferation potency in previous studies, and thus, no method for the real-time monitoring of metabolic activity at a fixed pH has been developed. Therefore, we attempted to establish a method for monitoring the acid-producing activity of cells in real time and to evaluate the direct effects of microenvironmental pH on the metabolic processes of cancer cells in comparison with normal cells.

Furthermore, to confirm that this monitoring system can also be applied to evaluate the effect of the anticancer agent, we attempted to measure the acid-producing activity from glucose in the presence and absence of 2-deoxy-D-glucose (2DG), one of anticancer agents which is known as a metabolic inhibitor^16^.

## Results

### Acid production from glucose, glutamine, and glutamate

**Figure 1:**
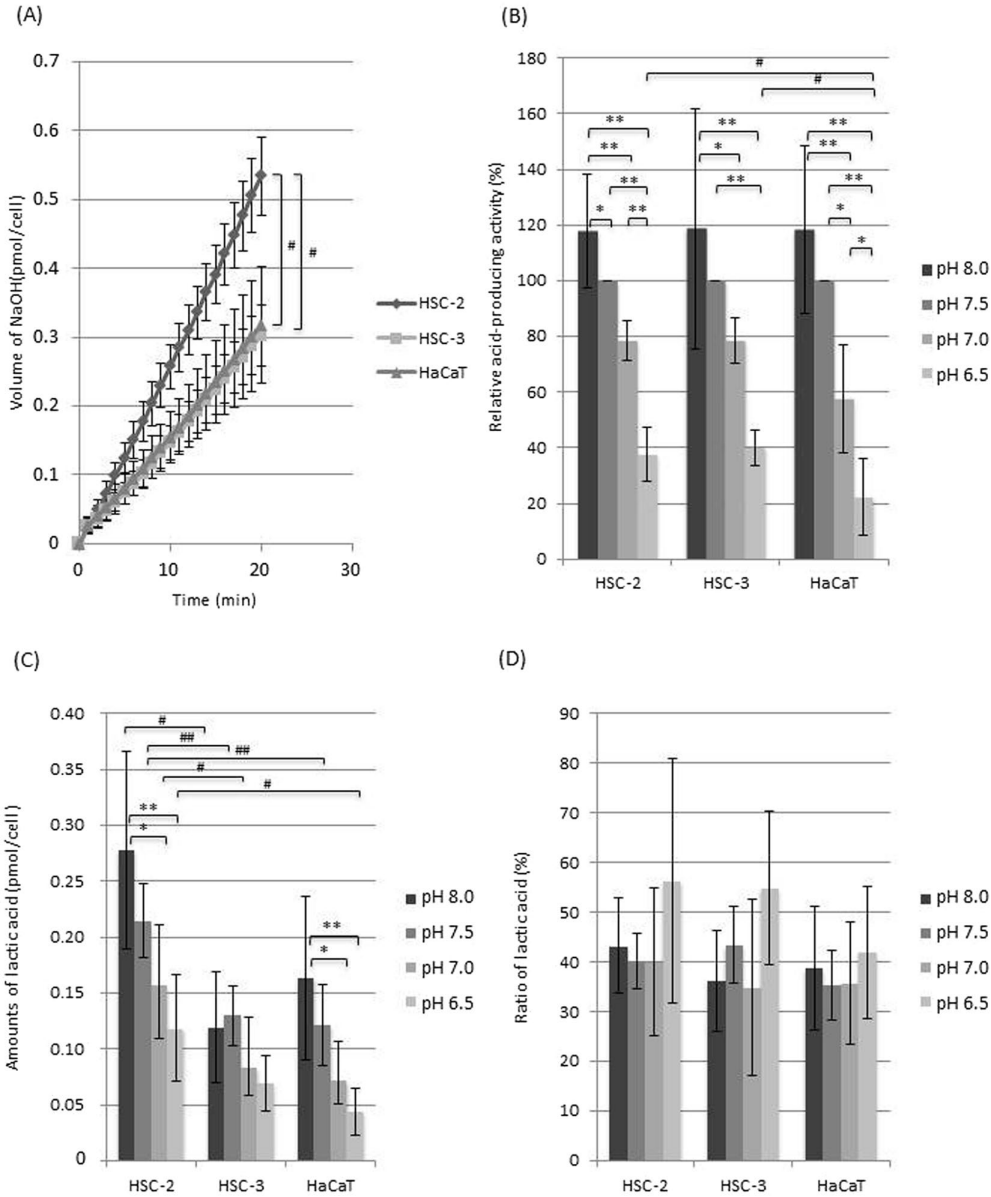
(**A**) Glucose-derived acid-production of each cell type at pH 7.5 (n = 5). The amount of NaOH indicates the amount of NaOH added during acid production from glucose after the glucose addition. Error bars represent standard deviations. ^#^Significant difference between each cells, p < 0.05 An ANOVA and Tukey’s test. (**B**) Relative glucose-derived acid-producing activity at various pH values (the activity at pH 7.5 was defined as 100%) (n = 5). Error bars represent standard deviations. *Significant difference between each pH condition, p < 0.05 An ANOVA and Tukey’s test. **Significant difference between each pH condition, p < 0.01 An ANOVA and Tukey’s test. ^#^Significant difference between each cells at the same pH condition, p < 0.05 An ANOVA and Tukey’s test. (**C**) Lactic acid production from glucose at various pH values (n = 5). The amount of lactic acid indicates the amount of lactic acid produced during acid production from glucose after the glucose addition. Error bars represent standard deviations. *Significant difference between each pH condition, p < 0.05 An ANOVA and Tukey’s test. **Significant difference between each pH condition, p < 0.01 An ANOVA and Tukey’s test. ^#^Significant difference between each cells at the same pH condition, p < 0.05 An ANOVA and Tukey’s test. ^##^Significant difference between each cells at the same pH condition, p < 0.01 An ANOVA and Tukey’s test. (**D**) The proportion of lactic acid among all acids at various pH values in the presence of glucose (n = 5). Error bars represent standard deviations.

**Figure 2:**
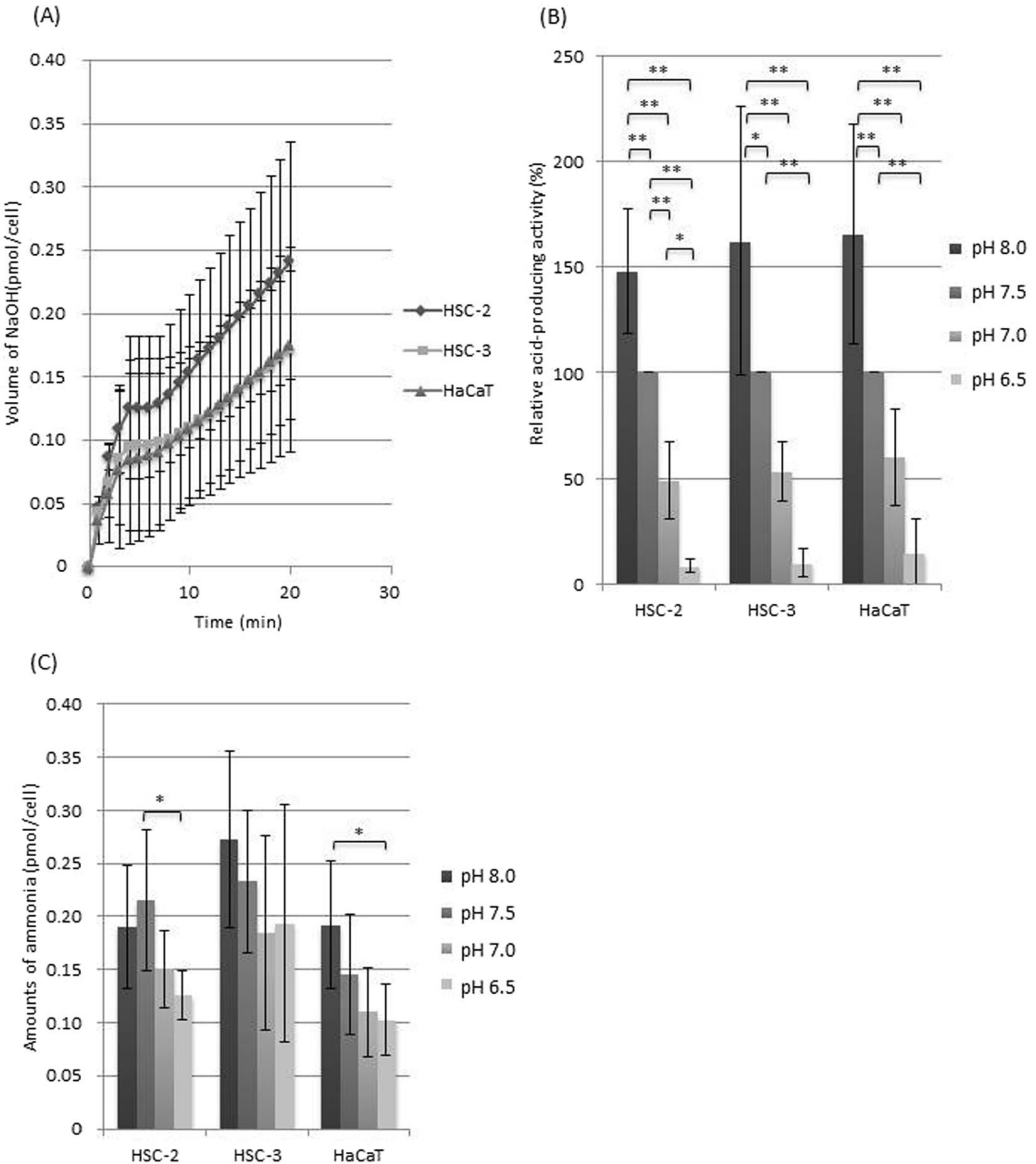
(**A**) Glutamine-derived acid-producing activity at pH 7.5 (n = 5). Error bars represent standard deviations. (**B**) Relative glutamine-derived acid-producing activity at various pH values (the activity at pH 7.5 was defined as 100%) (n = 5). Error bars represent standard deviations. *Significant difference between each pH condition, p < 0.01 An ANOVA and Tukey’s test. **Significant difference between each pH condition, p < 0.05 An ANOVA and Tukey’s test. (**C**) Ammonia production from glutamine at various pH values (n = 5). Error bars represent standard deviations. *Significant difference between each pH condition, p < 0.05 An ANOVA and Tukey’s test.

**Figure 3:**
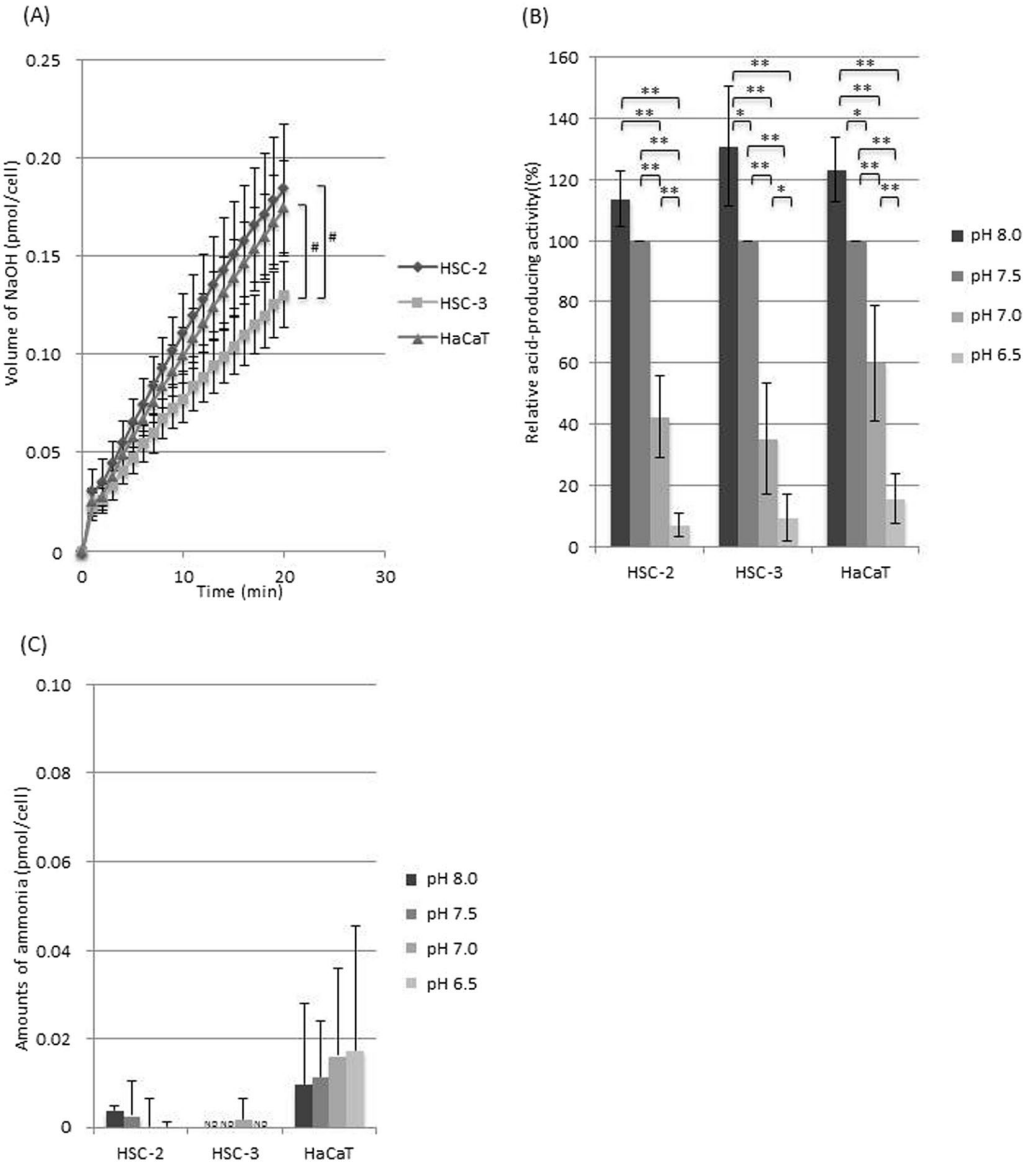
(**A**) Glutamate-derived acid-producing activity at pH 7.5 (n = 5). Error bars represent standard deviations. ^#^Significant difference between each cells, p < 0.05 An ANOVA and Tukey’s test. (**B**) Relative glutamate-derived acid-producing activity at various pH values (the activity at pH 7.5 was defined as 100%) (n = 5). Error bars represent standard deviations. *Significant difference between each pH condition, p < 0.05 An ANOVA and Tukey’s test. **Significant difference between each pH condition, p < 0.01 An ANOVA and Tukey’s test. (**C**) Ammonia production from glutamate at various pH values (n = 5). ND: not detected. Error bars represent standard deviations.

Both normal cells and cancer cells produced acids from glucose, glutamine, and glutamate. The acid-producing activity of the cells was successfully monitored in real time using a pH stat system. The amount of acid produced by the cells increased with time (Figs 1A–3A), and among the three metabolic substrates glucose induced the highest acid production rate. In the experiments involving glucose, the HSC-2 cells exhibited significantly greater acid production than the other cells (Fig. 1A).

### Effects of environmental pH on acid-producing activity

For each substrate, the acid production activity seen at pH 7.5 was defined as 100% and compared with those seen at other pH. In all cell types (regardless of the substrate used), acid production decreased as the environmental pH fell (Figs 1B–3B). A significant difference (p < 0.01) in acid production was observed between pH 8.0 or 7.5 and 6.5 in all cell types. The glucose-derived acid-producing activity of the cancer cells was more acid-tolerant than that of the HaCaT cells significantly (Fig. 1B), while the glutamine-derived acid-producing activity was similar among all the cell type (Fig. 2B) and the glutamate-derived acid-producing activity of the cancer cells seemed to be more acid-sensitive than that of the HaCaT cells (Fig. 3B).

### Lactic acid production from glucose

Both normal cells and cancer cells produced lactic acid from glucose, and the lactic acid production of the cells decreased as the environmental pH fell (Fig. 1C). The proportion of lactic acid among all acids (calculated from the amounts of NaOH titrated by the pH stat system) was about 35–55% (Fig. 1D). In the cancer cells the proportion of lactic acid among all acids tended to be higher than that of the HaCaT cells at acidic pH (pH 6.5).

### Ammonia production from glutamine and glutamate

All of the cells produced ammonia from glutamine (Fig. 2C), while the HaCaT cells produced ammonia from glutamate (Fig. 3C). Ammonia production from glutamine was reduced at acidic pH in all cell types.

### The effect of 2DG on the acid-producing activity of the HSC-2 cells

**Figure 4:**
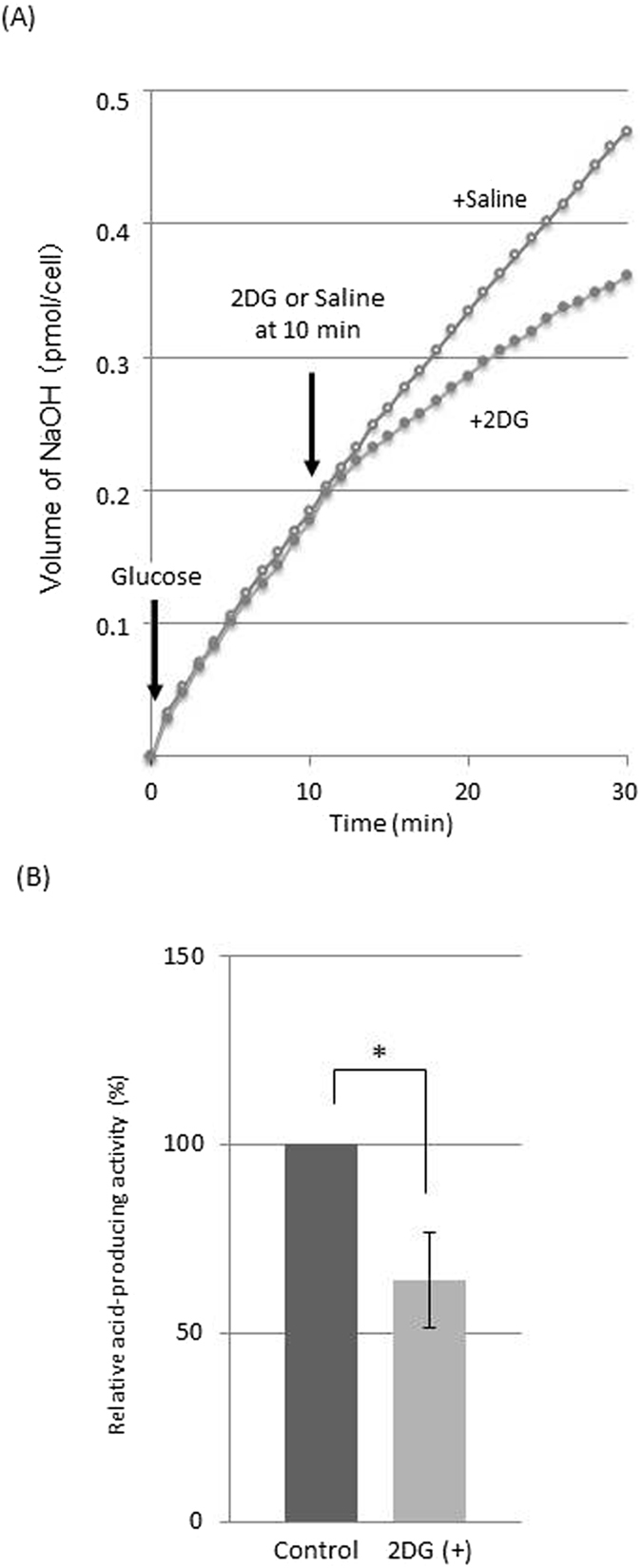
(**A**) An example of real-time monitoring of acid-producing activity from glucose and the effect of the 2DG addition at 10 min. (**B**) Relative acid-producing activity after the 2DG addition (the activity observed in control was defined as 100%) (n = 3). Error bars represent standard deviations. *Significant difference, p < 0.05 An ANOVA and Tukey’s test.

The addition of 2 mM 2DG at 10 min after glucose-induced acid production inhibited the acid-producing activity from glucose by the HSC-2 cells, while the addition of saline showed no effect (Fig. 4A). The inhibition% was 35.9 ± 12.8 (Fig. 4B).

## Discussion

In previous studies, the metabolic activity of cells has mainly been evaluated based on the expression of metabolic enzymes^12^, cell proliferation capacity^17^, and lactic acid production during the proliferation process^18^, etc. However, it is difficult to estimate real-time metabolic activity based on the gene expression of metabolic enzymes or lactic acid accumulation during cell proliferation.

**Figure 5:**
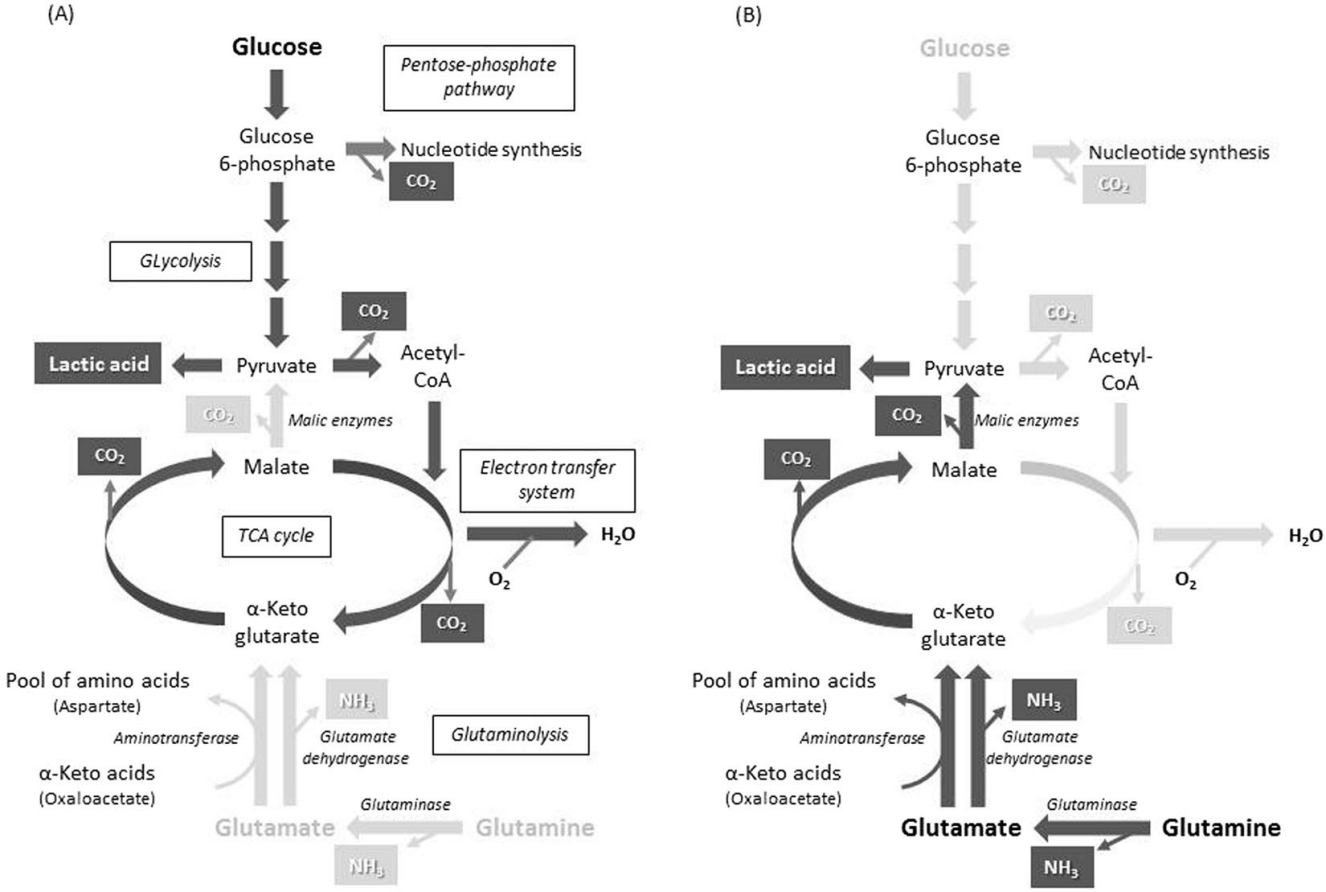
Proposed metabolic pathways in OSCC and normal cells. (**A**) Glucose metabolic pathways (dark lines). Main end-products are highlighted. Key metabolic enzymes are indicated in italic. (**B**) Glutamine and glutamate metabolic pathways (dark lines). Main end-products are highlighted. Key metabolic enzymes are indicated in italic.

In the present study, we successfully monitored the acid-producing activity of cancer and normal cells in the presence of glucose, glutamine, or glutamate at a fixed pH by continuously quantifying their acid production using a pH stat system (Figs 1A–3A). In general, glucose is metabolised via glycolysis, the TCA cycle, the electron transfer system, and the pentose phosphate pathway, and the products created during these processes are organic acids, such as lactic acid, CO_2_, and H_2_O (Fig. 5A). CO_2_ can dissolve in water, which results in its conversion to carbonic acid. Therefore, we can estimate the glucose-derived acid-producing activity of cells by monitoring the levels of these acids. All of the cells produced acids from glucose, and the amount of acid produced increased linearly with time (Fig. 1A), indicating that the cells metabolised glucose smoothly and that the acid-producing activity of cells can be estimated in real-time by monitoring their acid production.

It is generally accepted that glucose is metabolised to pyruvate via glycolysis, and pyruvate can be converted into lactic acid under anoxic conditions or further metabolised in the TCA cycle and electron transfer system with CO_2_ production under normoxic conditions. In addition, cancer cells are known to produce mainly lactic acid from glucose even under normoxic conditions (Warburg effect). Unexpectedly, the proportion of lactic acid among all acids, which was calculated from the amounts of NaOH titrated by the pH stat system, was 35–55%, and there was no significant difference in this parameter between the OSCC cells and the HaCaT cells (Fig. 1D). These findings suggest that the Warburg effect can occur in both cancer cells and normal cells. In recent years, the Warburg effect has been reexamined^19, 20^. Although it is well accepted that cancer cells possess a series of oncogene-directed metabolic reprogramming, which results in aerobic lactate production (Warburg effect)^21, 22^, it is also known that proliferating cells enhance glycolysis for rapid ATP production and macromolecular biosynthesis and subsequently produce lactate aerobically^23, 24^. In the present study, the HaCaT cells were collected at proliferating stage, so the cells might be able to express such metabolic properties.

At the acidic environment such as pH 6.5, the proportion of lactic acid in the OSCC cells tended to increase (Fig. 1D), suggesting that these cells shift the metabolic flow of glucose to lactic acid production in response to pH changes. The acid-producing activity of the HSC-2 cells was significantly greater than those of the other cell types (Fig. 1A). However, acid-producing activity can vary due to differences in cell lines and culture conditions, and therefore, it is difficult to generalise this phenomenon.

On the other hand, during the metabolism of glutamine or glutamate, ammonia was produced as well as acid (Figs 2C and 3C), and therefore, some of the acid produced would have been neutralised by the ammonia, which would have resulted in the underestimation of acid-producing activity. However, it was still possible to monitor the cells’ acid-producing activity (Figs 2A and 3A) in the presence of glutamine or glutamate, indicating that the cells were producing greater amounts of acid than ammonia from glutamine and glutamate, as expected in the glutaminolysis where glutamine/glutamate can be converted to lactic acid via the last half part of the TCA cycle and malic enzymes in OSCC cells^7^ and other cancer cells^25^ (Fig. 5B).

Acid production from glutamate increased linearly with time (Fig. 3A), while acid production from glutamine was rapid in the first 1–2 min, stopped for next few minutes, and then started again (Fig. 2A). Glutamine is considered to be metabolised to glutamate by glutaminase (Fig. 5B). This two-step reaction might result in pulsation; however, further study is needed to elucidate the underlying metabolic control mechanisms.

The HaCaT cells produced ammonia from both glutamine and glutamate, while the OSCC cells produced ammonia mainly from glutamine (Figs 2C and 3C). There was no difference in glutamine-derived metabolic activity between the OSCC cells and HaCaT cells, which suggested that the pathways involved in glutamate degradation differ between the cancer and normal cells. Glutamine is usually deaminated to glutamate, producing ammonia, by glutaminase, as stated above. Glutamate is further deaminated to α-ketoglutarate with the production of ammonia by glutamate dehydrogenase (Fig. 5B). In the normal cells, ammonia production was observed in the presence of glutamine or glutamate (Figs 2C and 3C), supporting the existence of these metabolic pathways. However, the absence of ammonia production from glutamate in the OSCC cells suggests that cancer-specific metabolic pathways also exist. Recently, it was demonstrated that in human pancreatic ductal adenocarcinoma cells glutamate is converted to α-ketoglutarate by aspartate aminotransferase, producing aspartate from oxaloacetate. Thus, glutamate could be used to maintain the pool of amino acids^26^. OSCC cells might possess a similar metabolic pathway involving aminotransferases which transfer glutamate-derived amino group to α-keto acids to form amino acids without ammonia production (Fig. 5B).

Extracellular acidosis is reported to be a feature of cancer tissue^8^. Hence, it is assumed that cancer cells have a greater capacity to adapt to low pH environments than normal cells. However, in the present study metabolic activity decreased as the environmental pH fell in all cell types regardless of the substrate present (Figs 1B–3B). The glucose-derived acid-producing activity of the cancer cells was more acid-tolerant than that of the HaCaT cells significantly (Fig. 1B), while the glutamine-derived metabolic activity was similar among all the cell type (Fig. 2B) and the glutamate-derived metabolic activity of the cancer cells seemed to be more acid-sensitive than that of the HaCaT cells (Fig. 3B). These observations suggest that differences in the acid sensitivity of metabolic pathways might exist between cancer cells and normal cells. Further study is needed to clarify these issues.

In the present study, the real-time monitoring method was applied to evaluate the inhibitory effect of 2DG, one of the anticancer reagents on the cancer cells. The inhibition of 2DG was clearly shown and quantified by this method (Fig. 5A,B), indicating that this method is suitable to evaluate the inhibitory effect of anticancer agents based on the metabolic activity.

In conclusion, the method established in the present study made it possible to monitor the acid-producing activity of cells in real time at a fixed pH. There are various existing methods/machines that enable to monitor cellular metabolic activity; however, one of the popular bioanalyzers measures pH decline during metabolism, so the reaction pH cannot be maintained, while measurement of cellular oxygen consumption during metabolism is not suitable to evaluate aerobic lactate production (Warburg effect) due to limit of oxygen utilization. In addition, the method using a pH-stat is inexpensive and easily set up under various conditions. This method might provide new topics for research into cancer cell metabolism and aid the evaluation of anticancer drugs. In most previous studies of anticancer drugs targeting metabolic enzymes in cancer cells, the drugs were mainly evaluated based on their ability to inhibit cell growth^27^. Our method can evaluate such inhibitory effects directly and could contribute to the establishment of more effective anticancer drug treatments.

## Materials and Methods

### Cell culture

Two lines of OSCC cells were used in this study. HSC-3 (RCB1975) cells, which are highly aggressive OSCC cells derived from the tongue, and HSC-2 cells (RCB1945), which are weakly aggressive OSCC cells derived from the mouth, were purchased from Riken Cell Bank (Tsukuba, Japan) and cultured as recommended. These cells were cultured in Eagle’s minimal essential medium (Wako Pure Chemical industires Ltd., Tokyo, Japan) supplemented with 2 mmol/L L-alanyl-L-glutamine solution (Wako Pure Chemical Industries Ltd., Tokyo, Japan), 10% heat-inactivated fetal bovine serum, 100 µg/mL streptomycin, and 100 U/mL penicillin at 37 °C with humidified 5% CO_2_ (CO_2_ incubator; model MCO-18AC, Sanyo, Tokyo, Japan). HaCaT cells (a human immortalised keratinocyte line derived from normal skin) were also purchased from Cosmo Bio (Tokyo, Japan) and used as normal cells. The HaCaT cells were cultured in Dulbecco’s modified Eagle’s medium (10-013-CVR, Corning, NY, USA) supplemented with 10% heat-inactivated fetal bovine serum, 100 mg/mL streptomycin, and 100 U/mL penicillin at 37 °C with humidified 5% CO_2_ (CO_2_ incubator; model MCO-18AC, Sanyo, Tokyo, Japan).

All of the cells were sub-cultured every 3–4 days to maintain logarithmic growth, collected at 80–90% confluence, and suspended in saline at a density of 1.0 × 10^6^ cells/ml. Before and after the experiment, the cells were confirmed to be alive using 0.4 w/v % trypan blue solution (Wako Pure Chemical Industries Ltd., Tokyo, Japan)^28^.

### Real-time monitoring of acid-producing activity with a pH stat system

After 10 min pre-incubation at 37 °C, the cell suspension was mixed with the relevant metabolic substrate (5 mM glucose, glutamine, or glutamate), and acid production was monitored for 20 min using a pH stat system (AUTO pH STAT; model AUT-211S, TOA Electronics, Tokyo, Japan) and 0.01 M NaOH as a titrant. When the pH of the reaction mixture began to fall due to acid production by the cells, NaOH was automatically added to the mixture when the pH became less than predetermined pH (6.5, 7.0, 7.5, or 8.0) by 0.01 pH unit, so every 2–10 second, 0.1 µL of NaOH was added and mixed in the reaction mixture to maintain the predetermined pH, and the amount of NaOH added was monitored by an integrator. The acid-producing activity of the cells was estimated from the amount of NaOH. All of the experiments were performed in normal air.

### Measurement of lactic acid and ammonia

At the start and end of the reaction, samples of the reaction mixture were collected and centrifuged immediately at 12,000 rpm for 2 min. The supernatant was used to measure the levels of lactic acid and ammonia. The supernatant was stored at −80 °C until the measurements were obtained.

After the supernatant had been pre-treated as reported previously^29^, the lactic acid level was measured using capillary electrophoresis-time-of-flight mass spectrometry (CE-TOFMS: G1600AX and G1969A; Agilent Technologies, Waldbronn, Germany). A fused silica capillary (H3305-2002; Human Metabolome Technologies) was used to separate out the metabolites during the CE. The applied voltage was set at −30 kV when the electrospray ionisation was performed in negative ion mode, and the capillary voltage was set at 3.5 kV in negative ion mode. The flow rate of the heated dry nitrogen gas (300 °C) was maintained at 7 L/min. The metabolites separated out by CE were mixed with sheath liquid (H3302-1020; Human Metabolome Technologies), which provides the electric contact as well as appropriate flow and solvent conditions for ensuring the optimal ionisation of the metabolites, and continuously sent to the TOFMS system for mass analysis.

Ammonia was measured with an ammonia meter (AMICHECK METER, Arkray, Kyoto, Japan), as reported previously^30^.

### Application of this real-time monitoring system to evaluate the inhibitory effect of anticancer agents

2-Deoxy-D-glucose (2DG; Wako) was used as a anticancer agent. The reaction mixture consisted of 1 mL of cell suspensions (1.0 × 10^6^ cells/mL) of the HSC-2 cells and 3.5 mL of saline. Firstly, this reaction mixture was set to the pH stat at pH 7.5 and pre-incubated at 37 °C with stirring for 10 min. Then, glucose (250 μL) was added to the reaction mixture at a final concentration of 10 mM, and their acid-producing activity was monitored for 10 min. At 10 min after the glucose addition, 2DG (250 μL) was added to the reaction mixture at a final concentrations of 2 mM and the acid-producing activity was further monitored for 20 min. Similarly, saline was added instead of the 2DG in control group.

### Statistical analysis

An ANOVA was performed and then Tukey’s test was employed to determine the statistical significance of the differences between the cancer cells and normal cells. P-values of < 0.05 were considered to be statistically significant.

The statistical comparison of the inhibition rates by 2DG was also analyzed using Tukey’s test. The data were analyzed using the Statflex Ver. 6 (Artech Co., Ltd., Osaka).
